# A Label-Free and Ultrasensitive Immunosensor for Detection of Human Chorionic Gonadotrophin Based on Graphene FETs

**DOI:** 10.3390/bios7030027

**Published:** 2017-07-12

**Authors:** Kamrul Islam, Ahmed Suhail, Genhua Pan

**Affiliations:** Wolfson Nanomaterials and Devices Laboratory, School of Computing, Electronics and Mathematics, Faculty of Science and Engineering, University of Plymouth, Drake Circus, Plymouth PL4 8AA, UK; ahmed.suhail@plymouth.ac.uk (A.S.); G.Pan@plymouth.ac.uk (G.P.)

**Keywords:** graphene, field effect transistor, immunosensor, human chorionic gonadotrophin

## Abstract

We report on a label-free immunosensor based on graphene field effect transistors (G-FETs) for the ultrasensitive detection of Human Chorionic Gonadotrophin (hCG), as an indicator of pregnancy and related disorders, such as actopic pregnancy, choriocarcinoma and orchic teratoma. Pyrene based bioactive ester was non-covalently anchored onto the graphene channel in order to retain the sp^2^ lattice. The G-FET transfer characteristics showed repeatable and reliable responses in all surface modifying steps using a direct current (DC) readout system. The hCG concentration gradient showed a detection limit of ~1 pg·mL^−1^. The proposed method facilitates the cost-effective and viable production of graphene point-of-care devices for clinical diagnosis.

## 1. Introduction

Human Chorionic Gonadotrophin (hCG) is a glycosylated protein (37.5 kDa) secreted by placenta during pregnancy or gestational trophoblastic diseases [1,2]. The function of hCG is to stimulate steroid hormone and progesterone production in the corpus luteum to support placental growth [3]. Hence, increased levels of hCG in the serum and urine are key indicators of trophoblastic tumors and other pregnancy related diseases [4,5,6]. As a result, it is significantly important to develop a highly selective and sensitive sensor to detect hCG for early diagnosis. At present, the detection methods for hCG mainly include various immunoassay based on electrochemical, electrochemiluminescence, fluorescence, enzyme, chemiluminescence resonance energy transfer, chemiluminescence and radiation [7]. Though these systems are sensitive and selective, but they tend to be expensive, time-consuming, labor intensive, bulky and require additional electrical system to translate into electrical signals. Hence, there is a need in manufacturing reliable and scalable electrical method to meet the demand for point of care testing (POCT) devices.

Since the first report by Bergveld et al. on FET based immunosensor (immunoFET), numerous studies have been conducted on immunoFET based on silicon nanowires (SiNW), SiO_2_ nanobelt, ZnO nanofilm, Au nanoparticles, molybdenum disulphide (MoS_2_), cerium oxide (CeO*_x_*) and single-walled carbon nanotubes (SWCNT) to name a few [8,9]. Recently, graphene attracted much interest by various research groups to focus on the possibility of graphene based FET (gFET) due to its unique characteristics regarding high surface to volume ratio, biocompatibility consisting of extreme sensitivity to the environment and ambipolar behavior [10,11,12,13]. Half a dozen of reports has appeared in last few years on back-gated graphene field effect transistor (BG-gFET) immunosensors, indicating that there is a growing interest from both academic and industrial researchers to meet all the challenges for cost-effective, easy to fabricate, scalable, reliable, ultrasensitive and rapid on-site detection devices [14,15,16,17,18,19]. Note that a non-covalent functionalization is of graphene is of much importance as covalent functionalization augments carrier scattering instigated by the conversion of planar sp^2^ to tetrahedral sp^3^ carbons leading to charge impurities and vacancy defects. To the best of our knowledge, there is only one report on BG-gFET based immunoassay for hCG detection, using multilayer epitaxial graphene grown on silicon carbide and a covalent functionalization method was used to anchor the necessary biomolecules. Other BG-gFET based immunoassays adopted intermediary modification of graphene either by hexamethyldisilazane (HMDS) or reduced to graphene oxide targeting rotavirus, DNA or cancer biomarkers [15,16,17].

In the present study, we demonstrate a scalable CVD grown monolayer graphene based FET immunosensor on a SiO_2_/Si substrate. A non-covalent bond between monolayer graphene and antibody was facilitated by applying a linker molecule known as 1-pyrenebutyric acid–N-hydroxysuccinimide ester (Pyr-NHS) via π-π bonding. The performance of the device was assessed under ambient conditions for over 10 devices to establish higher stability and repeatability. Changes in resistance difference between consecutive surface modifications displayed a distinctive pattern not commonly seen in relation to the usual increase in resistance owing to biomolecular absorption. The sensor exhibited an ultralow label-free detection limit of 1 pg·mL^−1^ with high sensitivity (0.30 Ω·ng^−1^·mL^−1^) and reproducibility (>87% yield). An in-depth analysis on carrier transfer for each surface modification was carried out for the first time for non-covalently functionalized BG-gFET illustrating the importance of net charge, amino acid content and the isoelectric point of biomolecules. These key factors are vital for bioelectronic sensing mechanism and device performance. The rapid, sensitive, selective, stable, repeatable and reliable detection suggests promising applications of bgFET structure for multiple marker detection on a point-of-care device.

## 2. Materials and Methods

### 2.1. Materials

Monolayer CVD graphene on Cu foil was purchased from Graphene Supermarket (Calverton, NY, USA). Photoresist 1805 G2, lift-off resist 3B (LoR) and their corresponding developer and remover were purchased from A-Gas Electronic Materials (Warwickshire, UK). All the other chemicals, such as Pyr-NHS, iron nitride, nitric acid, PBS, Poly (methyl methacrylate) (PMMA), Ethanolamine etc. at biochemical grade were purchased from Sigma Aldrich (Dorset, UK). Bovine serum albumin (BSA) at biochemical grade was purchased from Sigma Aldrich (Dorset, UK). 100 µg·hCG (ab126652) lyophilized powder and 1 mL of 0.02 mg·mL^−1^ complementary anti-hCG (ab8466) in PBS buffer were purchased from Abcam (Cambridge, UK) and the prepared aliquots were stored at −20 °C.

### 2.2. Characterization of BG-gFET

Raman spectra was collected in an XploRA Raman system (Horiba, Middlesex, UK) running a LabSpec 6.4.2.5 and equipped with a 532 nm HeNe laser delivering 20 mW of laser power at the sample. An Olympus BX41 microscope (Olympus Corp., Tokyo, Japan) with a 100 µm slit width, a 300 µm confocal hole, 1200 T grating and 1000× magnification was used. An MPlan N 100× microscope objective (N.A.: 0.90, W.D.: 0.21, F.N.: 22) focused the laser on the sample into a spot of ~7.45 µm diameter. A thermoelectrically cooled charge coupled device (CCD) camera was used for detection. Single spectra were collected for bare, coated and non-coated dentine by graphene from 1100 cm^−1^ to 3000 cm^−1^ range with five accumulations each lasting 15 s.

X-ray photoelectron spectroscopy (XPS) was performed with Kratos AXISULTRA with a mono-chromated Al kα X-ray source (1486.6 eV) using an emission current of 10 mA and an anode potential of 12 kV (120 W). The ULTRA was used in fixed analyzer transmission (FAT) mode, with pass energy of 80 eV for wide scans and pass energy 20 eV for high resolution scans. Cyclic Voltammetry (CV) measurements were carried out in 10 mM ferricyanide aqueous solution (1 M KCl solution) at room temperature with a scan potential range from 0.8 V to −0.4 V and a scan rate of 100 mV·s^−1^.

### 2.3. Sensor Test and Data Analysis

A Keysight B1500A semiconductor device parameter analyzer with an MPS150 probe station (Cascade Microtech GmbH, Thiendorf, Germany) was employed to investigate the electrical characteristics and sensing performance of the FET device at room temperature. The transistor measurement on the sensor was carried out by measuring the drain current (Ids) as a function of the gate voltage (Vg) (−100 V to +100 V) with a fixed drain-source voltage (Vds = 100 mV). The sensing signal of the device was recorded by monitoring the Ids as a function of drain-source voltage (Vd) (−1 V to +1 V) with a fixed gate voltage (Vds = 0 V). The data were analysed using SigmaPlot 13 software (Systat Software Inc, London, UK).

### 2.4. Wet Transfer of Graphene

**Figure 1:**
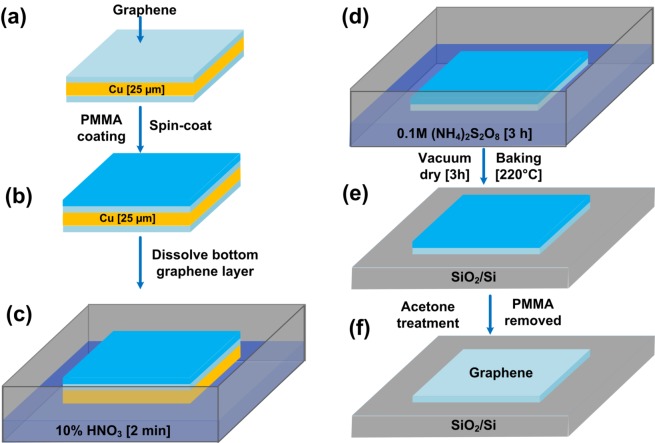
Schematic illustration of graphene wet transfer on SiO_2_/Si. The process begins with a graphene monolayer on Cu foil (**a**); After depositing a support layer composed of PMMA (**b**); the bottom graphene layer and Cu layer are etched by floating the sample on a HNO_3_ and (NH_4_)_2_S_2_O_8_ respectively (**c**,**d**); After transferring the resulting graphene-PMMA bilayer on a SiO_2_/Si substrate, PMMA removal by acetone (**e**,**f**) completes the transfer process.

The monolayer graphene was obtained from Graphene Supermarket (Calverton, NY, USA), and it was synthesized on both sides of 25 µm thick copper foil through chemical vapour deposition (CVD) method [20]. The wet transfer process as shown in Figure 1 was used to transfer monolayer graphene onto SiO_2_/Si substrates as follows. Firstly, PMMA was dissolved in the chlorobenzene with a 10 mg·mL^−1^ concentration and spin-coated on the one side of the graphene film at a spin speed of 4000 rpm for 30 s followed by baking at 180 °C for 1 min. To etch the Cu substrate, 10% HNO_3_ was used for 2 min followed by etching in 0.1 M ammonium persulfate for approximately 3 h with the endpoint determined when Cu was no longer visible. The resulted PMMA/graphene membrane was transferred to a rinse bath of deionized (DI)water for 10 min. Subsequently, monolayer graphene was directly transferred onto SiO_2_/Si substrates. Finally, PMMA layer was removed by acetone treatment at 50 °C for five minutes following by cleaning with Isopropyl alcohol (IPA) and DI water. Graphene on SiO_2_/Si substrates obtained as such was dried in vacuum before characterization and measurements.

### 2.5. Materials Fabrication of hCG Biosensor

**Figure 2:**
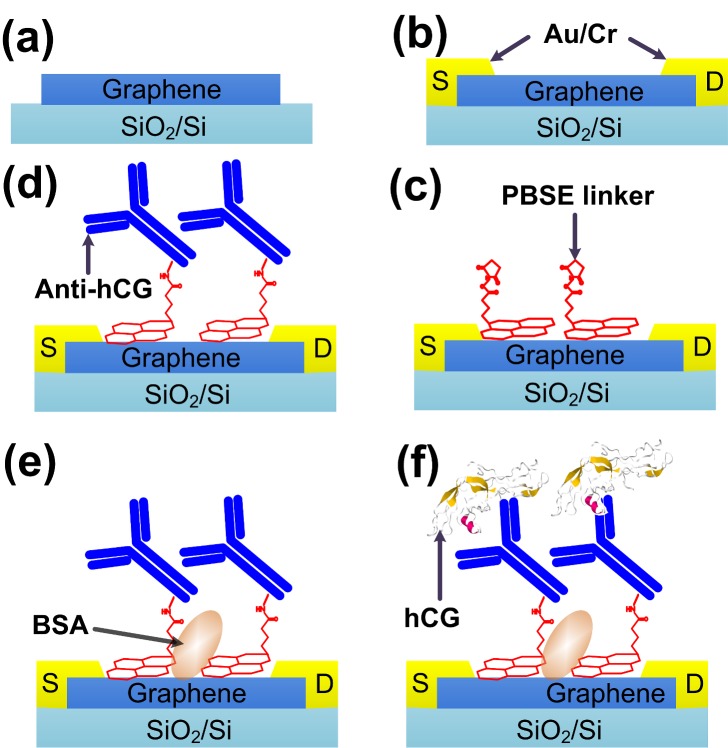
Schematic illustration of hCG biosensor fabrication. (**a**) monolayer graphene on SiO_2_/Si substrate; (**b**) Fabrication of BG-gFET; (**c**) anchoring of non-covalent linkers by π-π bond; (**d**) immobilization of antibodies; (**e**) blocking by BSA; (**f**) binding of hCG antigen.

A schematic illustration of the fabrication of hCG biosensor is shown in Figure 2. A flexible monolayer-layer graphene film on SiO_2_/Si substrate was obtained by wet-transfer method as mentioned above [21,22]. In order to fabricate source-drain and voltage electrodes, two consecutive photolithography was conducted. The lithography process begins with spin-coating LoR and positive photoresist (pPR) at 3000 rpm for 30 s. LoR was pre-baked at 175 °C for 15 min followed by soft-baking of spin-coated pPR at 100 °C for 1 min. For shaping graphene channel, samples were hard-baked at 180 °C for about an hour under deep UV (DUV) to reduce the pPR effect on graphene channel as much as possible [19]. We used a modified Argon plasma method to sputter the graphene on SiO_2_/Si at 50 W and 6 × 10^−7^ Torr for 2 min [23]. We deposited 5 nm Cr puddles for contact by thermal evaporator followed by 30 nm of Au layer by sputtering at 2 × 10^−7^ Torr and 4 mTorr base and Ar pressure [24]. The thickness of SiO_2_ is 300 nm and the width and length of each FET unit is 90 µm and 80 µm respectively.

Pyr-NHS was used as the linker between graphene channel and anti-hCG via π-π interaction. Consisting of hydrophobic pyrenyl moiety base and a bio-active ester head, Pyr-NHS does not require any pre-treatment of antigen with EDAC/NHS chemistry is required [14]. 2 mM Pyr-NHS solution was prepared by adding 0.0385 g Pyr-NHS powder into 5 mL methanol with modest shaking for 5 h. The linker solution was drop-casted on BG-gFETs and sealed in a moist environment for 2 h at room temperature, followed by methanol rinsing to avoid passivation layer formation. The functionalized graphene channels were covered by 10 µg·mL^−1^ anti-hCG solution and incubated at 4 °C for 4 h. The surface of graphene channel was rinsed with PBS buffer and immersed in BSA solution with a concentration of 0.5 mg·mL^−1^ to block the free amino groups to prevent the non-specific binding of hCG. Finally, hCG solution with concentrations ranging from 1 pg·mL^−1^ to 100 ng·mL^−1^ were prepared by diluting 1 µg·mL^−1^ stock solution with PBS buffer and then incubated on graphene channel at 4 °C for 4 h to ensure the strong antibody/antigen binding. Finally, the samples were treated with 100 mM ethnolamine for 30 min and washed with DIW for 5 min to remove non-specific passivation followed by vacuum dry at 30 Torr for an hour before characterization [25].

## 3. Results and Discussion

### 3.1. Assessment of Surface Morphology

**Figure 3:**
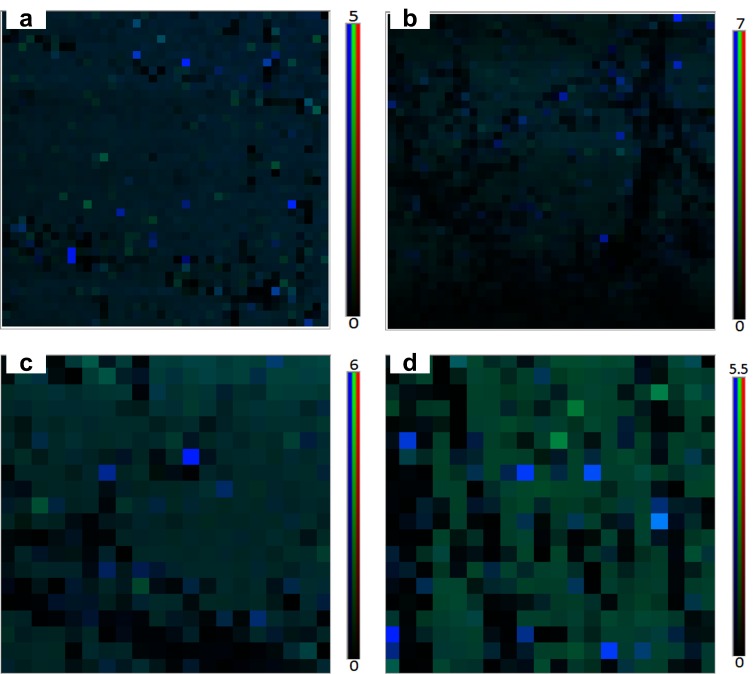
Raman maps of 2D/G for (**a**) bare graphene/SiO_2_; (**b**) linker + graphene/SiO_2_; (**c**) anti-hCG + linker/graphene/SiO_2_; (**d**) hCG + anti-hCG/linker/graphene/SiO_2_.

The morphology of the samples and surface modification was characterized by Raman spectroscopy, XPS and cyclic voltammetry. Figure 3 shows raman maps of 2D/G for graphene/SiO_2_ before and after adding linker and others. The intensity ratios of 2D-band to G-band are higher than 5. These data confirm that the transferred graphene before and after treating with linker and others is high-quality and continuous. For effective removal of photoresist during fabrication, deep UV (DUV) technique was used within the fabrication process to reduce photoresist contamination. The details of our work to obtain clean, uniform, and continuous graphene, with a typical low sheet resistance along with XPS and Cyclic voltammetry analysis have been elaborated in our recent works in detail [19,23]. The results demonstrated effective reduction of sheet resistance and contact resistance on the graphene surface by about 60% and 80%, respectively. Electrical current transport characteristics also demonstrated minimizing this residue on the graphene surface giving less hysteresis of electronic transport in BG-gFETs.

### 3.2. Qualitavie Assessment of Graphene and Surface Modification

**Figure 4:**
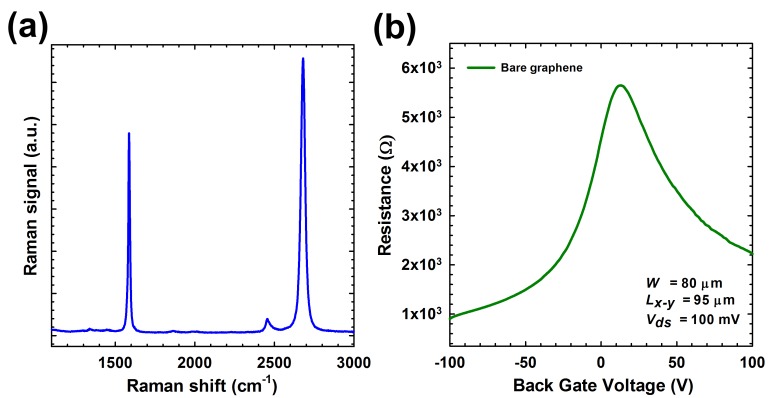
Raman characterization of patterned graphene channel (**a**) and resistance-back gate voltage curve of a typical graphene FET device (**b**).

Raman spectroscopy was used to confirm the layer number of graphene film. In the Raman spectrum shown in Figure 4a, the G-peak (~1600 cm^−1^) and 2D-peak (~2700 cm^−1^) are clearly resolved with strong intensity and sharp shape of 2D peak, indicating that the graphene film is monolayer. To avoid possible surface contaminations, all devices were thermal-annealed (Ar 1.5 ccm and H_2_ 250 sccm, 300 K) before electrical measurements were performed. Figure 4b presents the back gate (V_back·gate_)-dependent resistance measurement results of a typical graphene FET device, showing the ambipolar field-effect characteristics. Both the carrier type and density of graphene can be modulated by V_back·gate_. The maximum resistance (or the minimal conductance) point corresponds to the charge neutrality point V_cnp_ (or the Dirac point in the case of monolayer graphene), indicating how the graphene film is intrinsically doped, i.e., p/n doped when V_cnp_ is positive/negative or non-doped when V_cnp_ is equal to zero. For this particular device, V_cnp_ is around 12 V, suggesting it is intrinsically p-doped. The p-doping characteristics of graphene are usually caused by exposure to an oxygen-containing atmosphere, as oxygen molecules adsorbed onto defect or oxidized sites on a graphene surface will function as p-type dopants by trapping electrons during device fabrication process [26]. Several studies have shown that the conductance of graphene device exhibited ambipolar behavior and demonstrated better operating stability and higher sensing performance in the p-type region than in the n-type region [27,28]. The slight asymmetry around the Dirac point can be ascribed to the interaction of graphene with the contacts, as explained by Di Bartolomeo et al. [29,30].The feature of being sensitive to the surrounding environment renders graphene an ideal sensing material for detecting charged objects. With the presence of certain charged environment, the charge doping level (or Fermi level) of graphene will be changed, resulting in a shift of V_cnp_ or the resistance-V_back·gate_ curve.

### 3.3. Dependence of hCG Concentration

**Figure 5:**
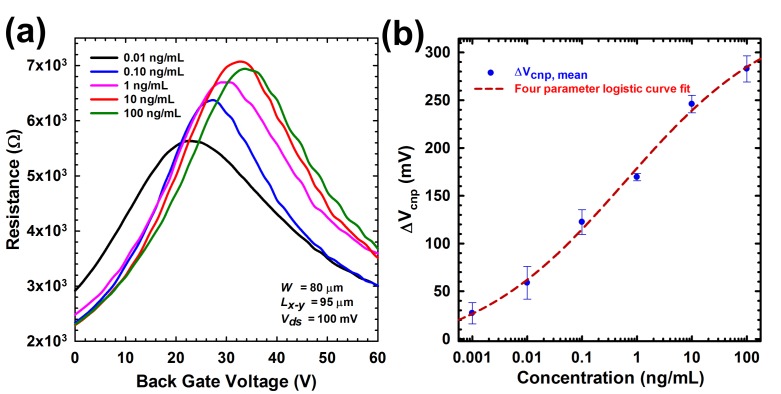
(**a**) The resistance-V_back·gate_ curves of five different hCG concentrations (1 pg·mL^−1^~100 ng·mL^−1^). (**b**) The measured ΔV_cnp_ versus concentration (in semilog scale); The red dotted line corresponds to the fitting results by using a four parameter logistic function.

To examine the performance of bgFET immunosensor for detection, we performed a quantitative analysis on multiple BG-gFET devices to examine the change in resistance of the graphene channel after Ab-hCG binding ranging from 1 pg·mL^−1^ to 100 ng·mL^−1^. All the devices were formed by modification processes under controlled physiochemical conditions. We used 100 µg·L^−1^ PBS to obtain standard solutions with different concentrations. After adding standard solutions with different concentrations for four hours at 4 °C followed by washing with PBS and N_2_ drying, we measured the resistance-V_back·gate_ curves within 2 min. The pH of standard solutions was kept at neutral to avoid false response. At least three replicates were conducted for each concentration and the relative standard deviations between replicates were 5%–24%, indicating good reproducibility of our measurement. The test results of standard solutions with five concentrations (i.e., 1 pg·mL^−1^, 10 pg·mL^−1^, 100 pg·mL^−1^, 1 ng·mL^−1^, 100 ng·mL^−1^) are shown in Figure 5a. Before adding solutions, the V_cnp_ was around 12 V, indicating the p-doping nature of the device in dry state. After adding standard solution of 1 pg·mL^−1^, we clearly observed right shifts of the curve, i.e., higher V_cnp_. For higher concentrations, the resistance-V_back·gate_ shifted further right and V_cnp_ became higher.

Such results indicate that our bgFET immunosensors are effective for detecting at different concentrations. The detection mechanism can be explained by a simple picture that, in dry condition, higher concentrations of hCG cause electron doping of the graphene (which is intrinsically p-doped) by approaching its surface, which leads to a right shift of the transfer curves or a higher V_cnp_ [31] and potentially used as an effective parameter to characterize ion concentrations. We tested multiple devices with similar response observed. In Figure 5b, we plotted the measured ∆V_cnp_ versus concentration (in logarithmic scale) of the tested standard solutions from three representative devices. A lognormal fitting was performed to obtain a calibration curve for quantification. Based on our measurement, the lowest concentration our graphene aptasensor detected was 1 pg·mL^−1^, and a concentration-response calibration curve showed a good fitting (R^2^ = 0.97) in the range of concentration evaluated, which is advantageous for the detection of hCG having wide-ranging expression profiles in the different stages of diagnosis i.e., pregnancy or cancer development. The obtained limit of detection (LOD) was 600 times more sensitive than the one achieved with the previously published hCG biosensor on BG-gFET [15] and twenty times better than one obtained with polysilicon nanogap [32]. In addition, our biosensor was capable of clearly monitoring the anti-hCG + hCG interaction at concentration as low as 1 pg·mL^−1^ directly without pre- or post-amplification process. This demonstrated that our aptasensor can work well for samples much lower than the safe level.

### 3.4. Slectivity, Stability, Reproducibility and Reusability

**Figure 6:**
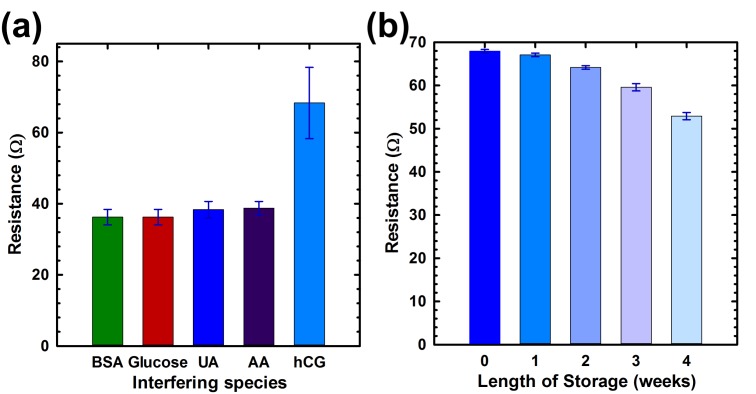
Selectivity and stability measurements for BG-gFET immunosensor. (**a**) The selectivity of the immunosensor with interfering species. BSA, Glucose (GLUC), Uric Acid (UA) and Ascorbic Acid (AA) were used; (**b**) long term stability of the biosensor for upto four weeks of storage.

Selectivity tests were carried out by using interfering species, such as BSA, glucose, uric acid (UA), ascorbic acid (AA) and 1 pg/mL of hCG (Figure 6a). It can be observed that the response of interfering species on our BG-gFET is less than 40 mV at the lowest concentration of hCG. In addition, the BG-gFETs showed satisfactory stability. After a month long storage, the resistance response decreased 21%, which is due to the gradual deactivation of the immobilized biomolecules (Figure 6b). At present, the sensor is for one-time use only, however, to extend the BG-gFET sensor into practical reusable applications, further work is needed.

## 4. Conclusions

In summary, we demonstrated a BG-gFET based immunosensor functionalized with a non-covalent linker for high sensitivity and label-free detection of hCG. The sensors showed the detection of hCG at concentration levels as low as 1 pg·mL^−1^, which is much lower than the clinical limit (5 ng·mL^−1^) with a broad dynamic range in concentration (1 pg·mL^−1^ to 100 ng·mL^−1^) [15]. Further development in multiple markers with a multiplex array is necessary for scalable and cost-effective detection. These results clearly show that BG-gFET immunosensors are promising devices for the use as label-free biological sensors that can electrically detect biomolecules.
